# Resilience and burnout among dual-role staff in a Chinese closed prison hospital during COVID-19

**DOI:** 10.1186/s40359-026-04910-x

**Published:** 2026-06-04

**Authors:** Cun-Bo Wu, Lei Gao, Fu-Xin Lin, Li-Chao Su, Zhang-Ya Lin

**Affiliations:** 1 Department of psychiatry, Chongqing mental health Center, Chongqing, 401147 China; 2 Department of Neurosurgery, The First Affiliated Hospital, Fujian Medical University, Fuzhou, 350005 China; 3 Neurosurgery Research Institute, The First Affiliated Hospital of Fujian Medical University, Fuzhou, 350005 China; 4 Department of Psychiatry, Shandong Ankang Hospital (Jining Mental Health Center), Jining, Shandong 272000 China

**Keywords:** COVID-19 pandemic, Quarantined Closed-Management, Job burnout, Resilience, Social support, General self-efficacy

## Abstract

**Objective:**

To deeply explore the resilience of job burnout among staff in a closed management prison hospital in China during the Coronavirus Disease 2019 (COVID-19) pandemic, to identify the influencing factors of resilience and its dynamic relationship with job burnout.

**Methods:**

A paper questionnaire survey was conducted. From June to July 2020, 200 prison staff from a prison hospital in Chongqing, China, were selected by random sampling method for the survey, and the results were statistically analyzed using SPSS statistical software.

**Results:**

There were significant differences in resilience between different prison wards, with the second ward having lower resilience than the administrative office, and the difference was statistically significant (*P* < 0.05); the second ward also had lower resilience than the third ward, and the difference was statistically significant (*P* < 0.05). Positive coping (β = 0.364, 95%CI:0.447 ~ 1.112) and General Self-Efficacy Scale(GSES) (β = 0.346, 95%CI:0.498 ~ 1.102) were positively correlated with resilience (*p* < 0.05). There was no correlation with psychoticism, social support, job burnout, prison wards, or neuroticism (*p* > 0.05).

**Conclusion:**

This study identified regional differences in Chongqing prison staff’s resilience, which correlates positively with positive coping and GSES but not directly with job burnout. Practically, target support for low-resilience units and integrate relevant training into routines to boost adversity adaptation. For prison hospital construction, address internal regional psychological impacts, establish job rotation and dynamic psychological assessment mechanisms, and optimize work environment and occupational security to build a “psychological support + institutional guarantee” model, informing national standardized development.

## Introduction

Against the COVID-19 pandemic, global society faced unprecedented trials — the medical and health sector served as the anti-epidemic frontline, while the prison system was also plunged into intense challenges. Reports show most Chinese prison staff adopted a closed-duty model: for example, all prisons in Guangdong implemented the highest-level duty system from 00:00 on February 22, 2020, with no rotation of on-duty police in supervision areas for nearly 2 months [1]. A survey indicates 72.9% of police officers deemed pandemic prevention risks, regulatory safety pressures, and standardized law enforcement demands beyond expectations [2], meaning their workloads far exceeded normal levels. For multi-role staff (doctors, nurses, special-duty police) at a Chongqing prison hospital, challenges were particularly acute. As a group with the dual identity of medical staff and prison personnel, they face the dual pressure of medical treatment and prison management for a long time, and such a special occupational characteristic makes the role of resilience particularly critical for them in resisting job burnout.

Relevant studies have confirmed that psychological resilience can effectively mediate the negative impact of work pressure on the mental health of prison police, who represent a typical high-stress occupational group with relatively low mental health levels [2]. For medical staff, psychological resilience has also been identified as a key factor in helping them cope with pandemic-related work pressure, such as shortages of protective materials and heavy diagnosis and treatment tasks [3]. However, existing studies mostly focus on single-identity groups such as prison police or ordinary medical staff, and lack targeted empirical research on the dual-identity group of prison hospital staff in public health emergencies, especially regarding their psychological resilience in response to increasingly prominent job burnout. This research gap makes it impossible to formulate precise psychological intervention strategies for this special group, which constitutes the core necessity of the present study.

Prison hospitals inherently possess their own distinctiveness, bearing the dual responsibilities of offering routine medical care and catering to the unique management requirements of the prison setting. They function within a year-round closed duty framework, devoid of mobile phones, computers, or other communication devices, and with restricted activities. The outbreak of the COVID-19 pandemic has exponentially increased their work pressure. Doctors must not only diagnose and treat potential COVID-19 cases with professional knowledge but also continuously monitor and treat a variety of other diseases, ensuring the quality of medical services under limited medical resources. Nurses have to stay on the front lines of care, performing high-intensity nursing procedures while strictly implementing various nursing protocols for epidemic prevention and control. As employees with the identity of police officers, in addition to maintaining the security and order of the prison, they must also assist the medical team in carrying out prevention and control work to ensure the stable operation of the prison during the pandemic. For this group, their occupational resilience is closely related to workplace support and pressure recovery ability. A two-year prospective cohort study on medical staff during the pandemic found that colleague support, workplace trust, and pressure recovery ability are key factors affecting occupational resilience [4]. This also implies that the closed working environment of prison hospitals, which limits external contact, may affect the formation of their resilience by weakening social support channels, which further highlights the necessity of exploring their resilience.

Employees who endure prolonged periods of high stress, risk, and uncertainty are often susceptible to job burnout. This condition is marked by emotional exhaustion, a tendency towards depersonalization in interactions with service recipients, and a pronounced sense of personal ineffectiveness [5]. Such experiences can have a detrimental effect on both the mental and physical well-being of employees, as well as their productivity at work. Research has shown that after over a year of continuous duty, numerous young prison police officers have developed psychological problems, including job burnout [6]. The mental health challenges faced by these young officers have been exacerbated by the extended periods of closed duty necessitated by the pandemic [7]. A study on Z-generation workplace groups also found that young employees have the lowest average psychological resilience, and long-term high-pressure closed work will further aggravate their job burnout and anxiety [8]. Some officers have reported being confined within the prison for up to nearly two months at the height of the pandemic [1], leading to significant weight loss once they were able to leave. These real-life data and anecdotes underscore the challenging conditions faced by prison police during the pandemic and the seriousness of job burnout issues, and also reflect the urgency of improving their psychological resilience.

Notably, even amid these challenging circumstances, some employees demonstrated strong adaptability, resisting the onset of job burnout to a certain extent and maintaining relatively positive work and psychological states. This resilience—defined as an individual’s ability to effectively cope with and recover from stress, adversity, and other challenges [9] —enables employees to reclaim work motivation and passion on the brink of burnout, allowing them to uphold their roles and fulfill their missions. Zhang Xiaomeng’s team’s research on the psychological state of groups during the pandemic pointed out that psychological resilience is negatively correlated with depression and anxiety, and improving resilience is an effective way to alleviate the psychological problems of employees under high pressure [10]. A prospective cohort study tracking Spanish medical staff for two years also confirmed that stress recovery ability and workplace trust can reduce the negative impact of pandemic pressure on employees by enhancing resilience [4]. Despite growing recognition of resilience’s importance, existing research suffers from three key limitations: it rarely focuses on the dual-identity group of prison hospital staff (medical professionals + prison personnel), lacks empirical data on resilience during public health emergencies like COVID-19, and fails to explore regional differences in resilience within prison systems or their underlying mechanisms. Addressing these gaps is central to the purpose of this study.

To achieve its core objectives, this research investigated the current levels of resilience and job burnout among staff across different positions (doctors, nurses, special-duty police) at the Chongqing prison hospital during the COVID-19 pandemic; analyzed variations in resilience based on demographic characteristics (e.g., age, work experience) and work-related factors (e.g., prison region, duration of closed duty); explored the relationship between resilience and key influencing factors, including personal traits, social support, organizational management, and coping strategies; clarified the dynamic correlation between resilience and job burnout to identify protective mechanisms against burnout.

A thorough exploration of these areas will not only illuminate the psychological and professional status of prison hospital staff under extreme conditions but also provide targeted, evidence-based recommendations for prison hospital management departments. Specifically, the findings will support efforts to enhance employees’ resilience, alleviate job burnout, and safeguard their mental health—ensuring that prison hospitals can operate efficiently and stably in future public health emergencies. As research on occupational resilience has proposed, building a supportive workplace culture and carrying out targeted resilience training are effective ways to improve employees’ ability to cope with crises [3, 4]. Ultimately, this research will protect the health and safety of prison inmates, maintain social stability, and underpin the long-term stable operation of the prison system, both during special periods and in daily operations. By focusing on this understudied group and context, the study aims to contribute novel empirical data to the field of occupational resilience and inform tailored support strategies for high-stress dual-role professionals.

## Objects and methods

A total of 200 staff members from a prison hospital in Chongqing, China, during the COVID-19 pandemic from June to July 2020, participated in this study.

### Measurement

The survey questionnaire included participants’ basic demographic information, such as gender, age, occupation, work environment during the COVID-19 pandemic, job satisfaction, job treatment, physical condition, job title, income situation, burnout, psychoticism, introversion-extraversion, neuroticism, and social support. Social support was measured using the Multidimensional Perceived Social Support Scale, and job burnout was measured using the Maslach Burnout Inventory-General Survey (MBI-GS). These scales have all previously been published in public. Details are as follows:

#### General situation questionnaire [10]

A self-designed instrument was employed to systematically collect respondents’ basic personal and occupational characteristics, including: ① Work-related variables: Prison ward (Ward 1/Ward 2/Ward 3/Administrative Office), department (Supervision Section/Medical Section/Administrative Section, etc.), years of service (≤ 5/6–10/11–15/>15 years), and professional title (Primary/Intermediate/Senior/None); ② Demographic variables: Age, gender, marital status, and education level; ③ Living and health-related variables: Income level, family structure, interpersonal satisfaction, and physical health status in the past six months. The questionnaire was validated through a pre-survey (*n* = 30), with a content validity index (CVI) of 0.89. As a categorical variable survey, no Cronbach’s α coefficient for internal consistency was required. This instrument provided baseline data for correlational analyses between demographic variables and core study variables.

#### Maslach Burnout Inventory-General Survey (MBI-GS) [11]

The Chinese version of the MBI-GS, revised by Li Chaoping et al., was adopted, which is adapted for Chinese occupational groups (including prison staff). In this study, the Cronbach’s α coefficients were as follows: Total scale = 0.85, indicating good internal consistency and reliability of the measurement tool. Emotional Exhaustion (5 items, e.g., “Work leaves me emotionally drained”) = 0.82; Depersonalization (4 items, e.g., “I feel indifferent to service recipients at work”) = 0.79; and Reduced Personal Accomplishment (6 reverse-scored items, e.g., “I effectively solve problems encountered in work”) = 0.81. A 5-point Likert scale was used (1 = Never to 5 = Always), with a total score range of 15–75. Occupational burnout was defined as a total score ≥ 50, or subscale cut-off values: Emotional Exhaustion ≥ 25, Depersonalization ≥ 10, and Reduced Personal Accomplishment ≤ 16. Higher scores indicated more severe levels of occupational burnout [11–13].

#### Eysenck Personality Questionnaire (EPQ) for adults [14]

The adult version of the EPQ, revised by Gong Yaoxian et al., was selected for this study. It is applicable to individuals aged ≥ 16 years and widely used in domestic personality research. In this study, the Cronbach’s α coefficients were: Total scale = 0.83; Extraversion (E scale, 21 items, e.g., “I enjoy participating in lively gatherings”) = 0.78; Neuroticism (N scale, 24 items, e.g., “I easily feel anxious and restless”) = 0.80; Psychoticism (P scale, 23 items, e.g., “I lack compassion for others”) = 0.76; and Lie scale (L scale, 20 items, e.g., “I always keep my words consistent with my deeds”) = 0.74. The scale consists of 88 items, using a dichotomous scoring method (Yes = 1 point, No = 0 point), with 26 reverse-scored items. Dimension interpretation: E scale scores (0–21) reflect the degree of extroversion and social initiative; N scale scores (0–24) indicate emotional instability (tendency toward anxiety, depression, and irritability); P scale scores (0–23) represent the inclination toward loneliness and aggression; L scale scores ≥ 10 suggest potential dissimulation or inaccurate responses, and such samples were excluded from the analysis. For the dichotomization of Psychoticism, Neuroticism: based on the norm standards of the EPQ scale, P scale score ≥ 12 was defined as “present”, < 12 as “absent”; N scale score ≥ 15 was defined as “present”, < 15 as “absent”.

#### General Self-Efficacy Scale (GSES) [15]

The Chinese version of the GSES, revised by Zhang Jianxin et al., was utilized to assess individuals’ confidence in coping with difficulties and challenges. In this study, the Cronbach’s α coefficient of the scale was 0.87, encompassing 10 items (e.g., “I can remain calm when facing difficulties because I trust my problem-solving abilities” and “Even when encountering troubles, I can find solutions”). A 4-point Likert scale was adopted (1 = Completely incorrect to 4 = Completely correct), with no reverse-scored items, and a total score range of 10–40. Scores were classified into three levels: low self-efficacy (10–20), moderate self-efficacy (21–30), and high self-efficacy (31–40). Higher scores reflected stronger confidence in overcoming adversity and greater stress tolerance.

#### Perceived Social Support Scale (PSSS) [16]

Based on the original scale developed by Zimet et al., the Chinese version revised by Jiang Qianjin was employed, which is adapted for assessing social support among occupational groups (correcting the prior bias of “being exclusively for patients”). In this study, the Cronbach’s α coefficients were: Total scale = 0.88; Family Support (4 items, e.g., “My family can understand my work pressure”) = 0.83; Friend Support (4 items, e.g., “I can obtain help from friends when encountering difficulties”) = 0.85; and Other Support (4 items, e.g., “My colleagues are willing to support me in work”) = 0.81. Good construct validity was confirmed by confirmatory factor analysis (CFA: χ²/df = 2.36, RMSEA = 0.07, CFI = 0.92). The scale comprises 12 items, using a 7-point Likert scale (1 = Strongly disagree to 7 = Strongly agree), with a total score range of 12–84. Scores were categorized as low-level support (12–36), moderate-level support (37–60), and high-level support (61–84). Higher scores indicated more sufficient perceived social support. For the dichotomization of Social Support: total score < 37 was defined as “hindered”, ≥ 37 as “unhindered”, based on the scale’s score classification standards.

#### Simplified Coping Style Questionnaire (SCSQ) [17]

The localized SCSQ, compiled by Xie Yaning, was used in this study. It is widely applied in domestic research on occupational stress and coping strategies, and adapted for high-pressure work settings of prison staff. In this study, the Cronbach’s α coefficients were: Total scale = 0.82; Positive Coping (12 items, e.g., “Confide my troubles to others” and “Proactively seek solutions to problems”) = 0.79; and Negative Coping (8 items, e.g., “Avoid problems that need to be addressed” and “Relieve stress through drinking/smoking”) = 0.76. A 4-point scoring method was adopted (0 = Not used to 3 = Frequently used), with a total score range of 0–60 (Positive Coping: 0–36; Negative Coping: 0–24). Higher scores on the Positive Coping dimension indicated a greater tendency toward adaptive coping strategies, while higher scores on the Negative Coping dimension suggested a stronger inclination toward maladaptive strategies.

#### Connor-Davidson Resilience Scale (CD-RISC) [18]

The Chinese version of the CD-RISC, revised by Zhang Jianxin et al., was designed specifically for high-pressure occupational groups (e.g., prison staff and medical workers). In this study, the Cronbach’s α coefficients were: Total scale = 0.91; Tenacity (13 items, e.g., “I can persist even when encountering setbacks”) = 0.88; Optimism (8 items, e.g., “I believe difficulties are temporary”) = 0.85; and Strength (4 items, e.g., “I can quickly recover from hardships”) = 0.83. The scale consists of 25 items, using a 5-point scoring method (0 = Never to 4 = Almost always), with a total score range of 0–100. Scores were classified into three levels: low resilience (0–30), moderate resilience (31–60), and high resilience (61–100). Higher scores indicated better psychological resilience and stronger adaptive capacity in response to occupational stress and emergencies.

### Steps

After signing the informed consent form and confirming their understanding of the study purpose, content, and relevant precautions, participants completed paper questionnaires. The study was conducted among staff members (including police officers, doctors, and nurses) from a prison hospital in Chongqing between June and July 2020. The target population comprised 200 eligible staff; 160 participants were selected using simple random sampling with the random number table method. A total of 160 questionnaires were distributed and collected, of which 145 were valid, with an effective response rate of 90.63%. These valid questionnaires provided reliable data for subsequent statistical analysis.

### Data analysis

Data analysis was conducted using SPSS22.0. Descriptive statistical methods were used to understand the demographic characteristics and the central tendency of the main variables. A series of ANOVA (Analysis of Variance) tests were conducted to compare the group differences in resilience between monitoring periods. Multiple linear regression analysis was used to study the predictive effect of epidemic-related factors on resilience. In addition, curve estimation analysis was performed to explore the nonlinear relationship between resilience and GSES, and the cubic curve model was fitted. The model fit indices such as R² change and F test for the cubic term were reported.

## Results

Out of 200 staff members, 160 cases were randomly (Using the random number table method) selected, with 145 valid responses, resulting in an effective response rate of 90.63%. A normality test was conducted on the resilience data with a sample size of 145. The Shapiro-Wilk test was used, and the *P*- value was 0.607 (> 0.05), indicating that the data approximately follow a normal distribution, meeting the preconditions for subsequent analysis.

Therefore, among these 145 individuals, Ward 1 has 23 (15.9%), Ward 2 has 59 (40.7%), Ward 3 has 20 (13.8%), and the administrative office has 43 (29.7%); doctors account for 34 (23.4%), nurses 24 (16.6%), and prison guards 87 (60%); the total sample includes 106 males (73.1%) and 39 females (26.9%); married 116 (80%), unmarried 24 (16.6%), divorced 5 (3.4%); in terms of education, secondary school 6 (4.1%), college 35 (24.1%), bachelor’s degree 100 (69%), and Master’s degree 4 (2.8%); work experience of less than 5 years 22 (15.2%), 6–10 years 45 (31%), 11–20 years 50 (34.5%), over 20 years 28 (19.3%); job titles include junior 67 (46.2%), intermediate 67 (46.2%), associate senior 10 (6.9%), and senior 1 (0.7%); family satisfaction is unsatisfied 4 (2.8%), less satisfied 12 (8.3%), average 33 (22.8%), more satisfied 48 (33.1%), and satisfied 46 (31.7%); interpersonal relationships are unsatisfied 3 (2.1%), less satisfied 7 (4.8%), average 51 (35.2%), more satisfied 48 (33.1%), and satisfied 36 (24.8%); income satisfaction is unsatisfied 17 (11.7%), less satisfied 19 (13.1%), average 68 (46.9%), more satisfied 27 (18.6%), and satisfied 14 (9.7%); physical condition is unsatisfied 14 (9.7%), less satisfied 25 (17.2%), average 59 (40.7%), more satisfied 36 (24.8%), and satisfied 11 (7.6%); work environment is unsatisfied 15 (10.3%), less satisfied 21 (14.5%), average 60 (41.4%), more satisfied 35 (24.1%), and satisfied 14 (9.1%); job treatment is unsatisfied 9 (6.2%), less satisfied 22 (15.2%), average 78 (53.8%), more satisfied 24 (16.6%), and satisfied 12 (8.3%); job burnout is present in 35 (24.1%) and absent in 110 (75.9%); no psychopathy in 111 (76.6%) and present in 32 (22.4%); no neuroticism in 68 (46.9%) and present in 75 (51.7%); extroverted 54 (37.2%), neutral 30 (20.7%), introverted 59 (40.7%); social support is hindered in 52 (35.9%) and unhindered in 92 (63.4%). The specific demographic characteristics of the participants are shown in Table 1.

**Table 1:** Respondent characteristics (*N* = 145)

Characteristic		Frequency	Percent(%)	Missing
Prison ward				0
	Ward 1	23	15.9	
	Ward 2	59	40.7	
	Ward 3	20	13.8	
	administrative office	43	29.7	
Occupation				0
	Doctor	34	23.4	
	Nurse	24	16.6	
	Prison guard	87	60	
Gender				0
	Male	106	73.1	
	Female	39	26.9	
Marriage				0
	Married	116	80	
	Unmarried	24	16.6	
	Divorced	5	3.4	
Educational level				0
	Secondary vocational school	6	4.1	
	Associate degree	35	24.1	
	Undergraduate	100	69	
	Master’s degree	4	2.8	
Years of work experience				0
	less than 5 years	22	15.2	
	6–10 years	45	31	
	11–20 years	50	34.5	
	over 20 years	28	19.3	
Title				0
	junior	67	46.2	
	Intermediate	67	46.2	
	associate senior	10	6.9	
	senior	1	0.7	
Family situation				0
	unsatisfied	4	2.8	
	less satisfied	12	8.3	
	average	33	22.8	
	more satisfied	50	34.5	
	satisfied	46	31.7	
Interpersonal relationships				0
	unsatisfied	3	2.1	
	less satisfied	7	4.8	
	average	51	35.2	
	more satisfied	48	33.1	
	satisfied	36	24.8	
Income situation				0
	unsatisfied	17	11.7	
	less satisfied	19	13.1	
	average	68	46.9	
	more satisfied	27	18.6	
	satisfied	14	9.7	
Physical condition				0
	unsatisfied	14	9.7	
	less satisfied	25	17.2	
	average	59	40.7	
	more satisfied	36	24.8	
	satisfied	11	7.6	
Work environment				0
	unsatisfied	15	10.3	
	less satisfied	21	14.5	
	average	60	41.4	
	more satisfied	35	24.1	
	satisfied	14	9.7	
job treatment				0
	unsatisfied	9	6.2	
	less satisfied	22	15.2	
	average	78	53.8	
	more satisfied	24	16.6	
	satisfied	12	8.3	
job burnout				0
	absent	110	75.9	
	present	35	24.1	
Psychoticism				2
	absent	111	76.6	
	present	32	22.4	
Extroverted-introverted				2
	introverted	59	40.7	
	neutral	30	20.7	
	extroverted	54	37.2	
neuroticism				2
	absent	68	46.9	
	present	75	51.7	
Social support				1
	hindered	52	35.9	
	unhindered	92	63.4	

The missing data in Table 1 is due to the fact that some subjects did not fill in the corresponding items when completing the questionnaire, and the missing rate is low (less than 2%), which does not affect the overall analysis results

The results of the analysis of variance indicate that there is a statistically significant difference in resilience among staff members between different departments (F = 2.890, *p* = 0.038 < 0.05), with the second detention area having lower resilience compared to the administrative office, and the difference is statistically significant (*P* < 0.05); the second detention area also has lower resilience compared to the third detention area, and the difference is statistically significant (*P* < 0.05). See Table 2.

**Table 2:** Multiple comparisons of ANOVA for resilience in different wards

Prison ward	Prison ward	MD	SE	*P*
Ward 1	Ward 2	1.564	3.701	0.673
	Ward 3	-6.426	4.603	0.165
	administrative office	-6.128	3.889	0.117
Ward 2	Ward 1	-1.564	3.701	0.673
	Ward 3	-7.990*	3.895	0.042
	administrative office	-7.692*	3.019	0.012
Ward 3	Ward 1	6.426	4.603	0.165
	Ward 2	7.990*	3.895	0.042
	administrative office	0.298	4.075	0.942
administrative office	Ward 1	6.128	3.889	0.117
	Ward 2	7.692*	3.019	0.012
	Ward 3	-0.298	4.075	0.942
*F*		2.890 *		0.038

* The mean difference is significant at the 0.05 level

As shown in Table 3, resilience has a weak positive correlation with negative coping (*r* = 0.215 < 0.3), prison ward (*r* = 0.203 < 0.3) (*p* < 0.05), and a strong correlation with positive coping (*r* = 0.597, *r* > 0.5), GSES (*r* = 0.534, *r* > 0.5) (*p* < 0.05). It has a moderate positive correlation with social support (*r* = 0.390, 0.3 < *r* < 0.5) (*p* < 0.05), and a weak negative correlation with neuroticism (*r*=-0.274 < 0.3), psychoticism (*r*=-0.297 < 0.3), and job burnout (*r*=-0.249, *r* < 0.3) (*p* < 0.05). There is no correlation with extraversion-introversion, job treatment, work environment, physical condition, age, occupation, gender, marital status, education level, job title, work experience, monthly income, family situation, and interpersonal relationships (*P* > 0.05).

**Table 3:** Correlation between resilience and various factors

	Negative coping	positive coping	GSES	Social support	neuroticism	extraversion-introversion	psychoticism	job burnout	job treatment	work environment	physical condition	Income situation	Prison ward	age	occupation	gender	Marriage	education level	job title	work experience	monthly income	family situation	interpersonal relationships
R	0.215**	0.597**	0.534**	0.390**	− 0.274**	0.128	− 0.297**	− 0.249**	0.099	0.087	0.028	− 0.004	0.203*	− 0.070	− 0.003	0.083	0.108	0.146	0.019	− 0.038	0.079	− 0.037	− 0.055
P	0.009	0.000	0.000	0.000	0.001	0.126	0.000	0.003	0.235	0.297	0.737	0.966	0.014	0.523	0.970	0.321	0.195	0.080	0.820	0.651	0.346	0.661	0.513

*P < 0.05, **P < 0.01

As shown in Table 4, the samples are independent of each other (D-W = 1.807), the model fit is moderate (Adjusted R2 = 0.479), and there is no multicollinearity (VIF < 5). After excluding the interference of confounding factors, positive coping and GSES are independent protective factors affecting resilience, among which positive coping (β = 0.364, 95%CI:0.447 ~ 1.112) and GSES (β = 0.346, 95%CI:0.498 ~ 1.102) are positively correlated with resilience (*p* < 0.05). They are not related to psychoticism, social support, job burnout, prison area, and neuroticism (*p* > 0.05).

**Table 4:** Multiple Linear Regression Analysis of Factors Associated with Resilience

Model			β	t	*p*	95.0% Confidence Interval for B			
	B	SE				Lower Bound	Upper Bound	Tolerance	VIF
(Constant) positive coping negative coping social support Psychoticism Neuroticism GSES prison area job burnout	4.358	6.930		0.629	0.531	-9.349	18.065		
	0.779	0.168	0.364**	4.641	0.000	0.447	1.112	0.597	1.676
	0.191	0.225	0.060	0.848	0.398	− 0.254	0.635	0.737	1.357
	0.143	0.080	0.128	1.781	0.077	− 0.016	0.302	0.706	1.416
	− 0.773	0.518	− 0.101	-1.493	0.138	-1.797	0.251	0.799	1.252
	− 0.085	0.320	− 0.019	− 0.266	0.791	− 0.718	0.548	0.726	1.378
	0.800	0.153	0.346**	5.234	0.000	0.498	1.102	0.838	1.193
	− 0.175	0.966	− 0.012	− 0.181	0.857	-2.085	1.735	0.800	1.250
	-1.855	2.365	− 0.052	− 0.784	0.434	-6.533	2.824	0.850	1.177
F	17.288*				0.000				
Adjusted R^2^	0.479								
D-W	1.807								

‘-’ indicates no data, *β* standardized coefficient, *SE* standard error

*P < 0.05, **P < 0.01

**Fig. 1:**
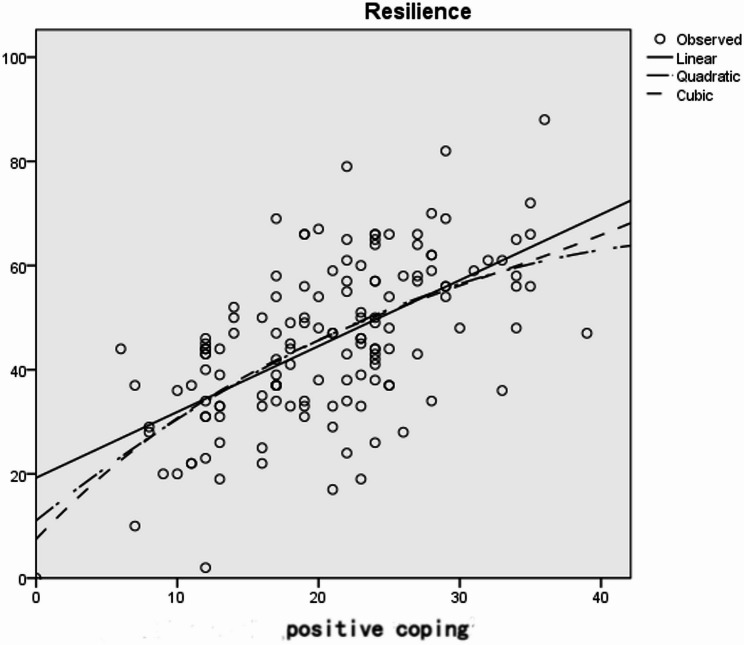
The Relationship Between Resilience and Positive Coping. Resilience increases with the increase in the score of positive coping

**Fig. 2:**
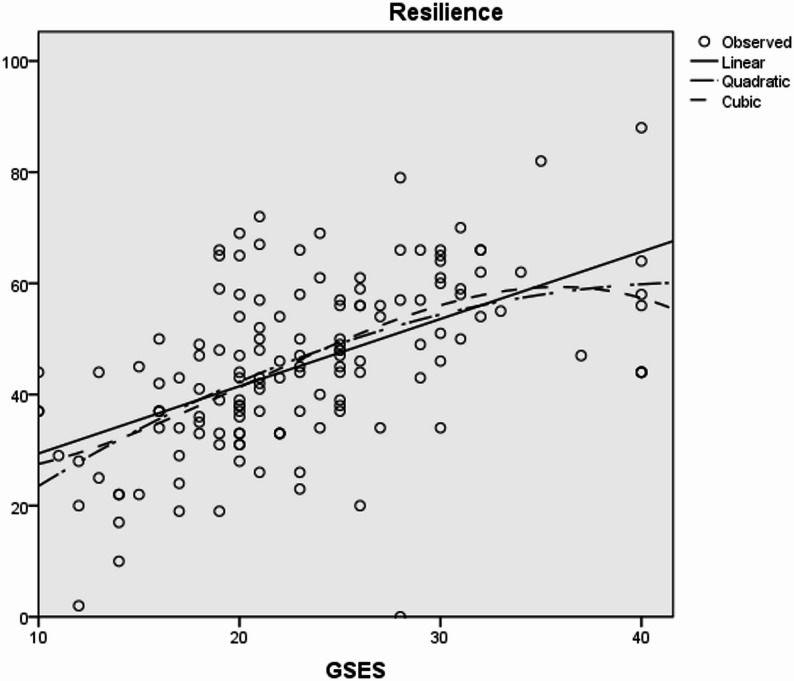
The relationship between resilience and GSES. Resilience shows a nonlinear relationship with GSES, increasing initially then decreasing at very high GSES scores. The results of curve estimation analysis show that the cubic curve model fits well (R² change = 0.032, F = 4.567, *p* = 0.004), indicating that the cubic term has a significant contribution to the model

From the curve of resilience, it can be seen that resilience increases with the increase in the score of positive coping, as shown in Fig. 1. Resilience shows a nonlinear relationship with GSES, increasing initially then decreasing at very high GSES scores, as shown in Fig. 2.

## Discussion

This study conducted an in-depth investigation into the resilience of prison staff in Chongqing, China, against job burnout. The findings not only reveal significant differences in resilience between different prison areas but also clarify the close relationship between resilience, social support, general self-efficacy (GSES), and job burnout. The following will discuss these important findings in detail, analyze the potential limitations of this study, and specifically address the differences between different prison zones as required.

### Differences in resilience and potential reasons across different prison zones

Research has found that the resilience of staff in the second zone of a prison in Chongqing, China, is significantly lower than that of the administrative department, while the resilience of staff in the third zone is significantly higher than that of the second zone, and the differences are statistically significant. There may be various factors behind this phenomenon.

The First Prison District Accounts for 15.9% (23/145) of the sample, mainly manages non-ill prisoners such as nursing prisoners, handyman prisoners, medical civilian prisoners, and strictly managed prisoners, nurses focus on basic health management for non-ill incarcerated individuals (including daily health rounds, common disease prevention, simple wound care, and health record maintenance). Their pressure stems from supervision staff’s consultations on nursing collaboration and the update of inmates’ health information, with moderate overall pressure due to the simple health conditions of service recipients and fixed workflows.

the Second Prison District accounting for 40.7% (59/145), mainly manages sick prisoners including those undergoing hemodialysis, with internal diseases, in critical conditions, and with infectious diseases, nurses undertake full-process professional nursing for ill incarcerated individuals (such as hemodialysis care, internal disease management, critical care, and infectious disease prevention and control). Pressure is concentrated on dual-responsibility conflicts, sudden changes in patients’ conditions, infection risks, and high-intensity nursing workloads—these are the core occupational factors leading to low resilience among Ward 2 staff.

the Third Prison District Accounts for 13.8% (20/145), mainly manages mentally ill prisoners; and the authorities are mainly responsible for second-line administrative work without directly managing prisoners, nurses specialize in mental health nursing for incarcerated individuals with mental illnesses (including symptom observation, medication supervision, psychological assessment, and crisis intervention support). Pressure arises from the unpredictability of inmates’ behaviors, high requirements for professional skills, and the urgency of crisis response, which is effectively mitigated by regular specialized training. There are no full-time nurses in the Administrative Office (29.7% of the sample); relevant health support work is undertaken by frontline nurses on a part-time basis (including staff health consultations and guidance on simple stress management skills), with low pressure and no direct risk exposure.

The second zone, as the front line of prison management, bears the high-intensity and high-pressure tasks of directly supervising inmates. The daily management of inmates and the response to emergencies make the staff in the second zone under a state of high tension for a long time. Statistical results confirm that Ward 2 has significantly lower resilience than Ward 3 (MD=-7.990, *p* = 0.042) and the Administrative Office (MD=-7.692, *p* = 0.012), which is consistent with Lambert et al. conclusion that “work environments with dual responsibilities (supervision + medical care) significantly increase staff emotional exhaustion and reduce resilience”, Recent studies have shown that long-term exposure to such high-pressure environments can easily lead to psychological fatigue and emotional exhaustion [19], which is closely related to the occurrence of job burnout, thereby affecting their resilience levels. In contrast, the administrative department is mainly responsible for administrative management and coordination, with a relatively stable work environment and relatively single sources of stress. Employees can perform their duties in a relatively relaxed atmosphere without direct contact with prisoners, which may help maintain higher resilience [20].

The resilience of the third ward is higher than that of the second ward, which may be attributed to its effective measures in team building and stress management. For example, the third ward may have created a more harmonious team atmosphere, with closer support and collaboration among employees, and more humane and approachable leadership. According to foreign studies, a positive team atmosphere can enhance employees’ sense of belonging and psychological resilience, effectively improving their resilience to stress [21]. In recent years, there have also been many research findings on the psychological conditions of prison staff. For instance, Skeaff [22] et al. found in their investigation of the mental health of staff in a provincial prison system that there are differences in the degree of job burnout and resilience levels among employees in different job positions, and that the job burnout situation of front-line ward staff is relatively more severe. Although this study did not explicitly involve the same regional comparison as the current study, it also reflects the impact of job differences on employees’ psychological conditions, providing us with some reference for understanding the situation of employees in different wards in our study. In a large-scale survey study of prison staff, found that the closed and high-pressure nature of the work environment is closely related to employees’ job burnout and resilience [23]. They pointed out that employees in front-line positions like the second ward, who are directly facing the inmates, are more likely to experience symptoms of job burnout due to being in a closed and high-pressure environment for a long time, and their resilience levels are relatively low, which is highly consistent with the results of this study regarding the resilience of employees in the second ward.

### Exploration of the relationship between resilience, positive coping and GSES

#### The impact of positive coping on resilience

Resilience is positively correlated with proactive coping, a result that aligns with the conclusions of many related studies. Proactive coping provides support for the resilience of prison staff in various ways. Proactive coping strategies help prison staff actively adopt effective strategies to resolve difficulties when facing occupational stress, thereby continuously strengthening their resilience in the process of coping. A higher sense of self-efficacy means that employees have a stronger confidence in their ability to successfully cope with various work challenges, and this positive self-perception will make them more resilient in the face of stressful situations, thereby enhancing their resilience level [24]. Proactive coping is an individual’s behavioral pattern when facing stress and challenges, characterized by taking active and constructive approaches to deal with problems, regulate emotions, and maintain psychological balance [23, 25]. When prison staff have higher resilience, they are more inclined to view difficult situations at work with a positive perspective, such as the management challenges of inmates, high-intensity workloads, and relatively closed and oppressive work environments. This positive mindset prompts them to actively seek effective coping strategies, rather than passively endure or avoid stress.

Many studies on high-stress occupational groups (such as medical staff, teachers, etc.) have also found a positive correlation between resilience and proactive coping [26]. For example, medical staff facing the intense tasks of medical treatment and the pressure of doctor-patient relationships, those who can cope proactively often show higher resilience and can recover more quickly from work stress, maintaining a good psychological state. This indicates that proactive coping is one of the key factors in enhancing individual resilience, regardless of the high-stress profession.

However, due to the uniqueness of the prison work environment, the results of this study also have their particularities. Prison staff not only have to face the intensity and pressure of work but also have to deal with the potential safety risks and psychological challenges posed by the special group of inmates. Unlike other professions, the stress situations in prison work are often more complex and sensitive, requiring higher psychological resilience from employees. Therefore, the interaction mechanism between resilience and positive coping in the prison staff population may be more complex, and the degree of association may be influenced by more factors, such as prison safety management measures and the effectiveness of education and reform for inmates. This also suggests that in subsequent studies, we need to further explore the specific impact of these special factors on the relationship between the two.

Additionally, one research emphasizes the importance of individual psychological traits such as optimism and resilience in building resilience among prison staff, similar to the positive correlation between positive coping and self-efficacy and resilience involved in this study [27]. For example, someone found in a survey study of prison staff in several prisons in the United States that a positive coping style plays a crucial role in alleviating occupational stress and enhancing psychological resilience among prison staff. The study points out that employees who can actively adopt positive coping strategies show stronger psychological resilience, that is, resilience, when facing stress situations such as prison violence incidents and inmates’ psychological crises. This is highly consistent with the conclusion of the positive correlation between resilience and positive coping in this study, further corroborating the importance of positive coping for enhancing resilience among prison staff [28, 29]. Strohmeier found through long-term follow-up studies of some prisons in Europe that the cultivation of resilience among prison staff is not only dependent on individual characteristics but is also closely related to external factors such as the prison’s organizational culture and management model. Among them, a positive organizational culture can encourage employees to adopt positive coping methods to deal with problems at work, thereby promoting the overall enhancement of staff resilience [30].

#### The impact of GSES on resilience

General Self-Efficacy Scale (GSES) is positively correlated with resilience, highlighting the importance of employees’ beliefs in their own abilities.

Employees with high GSES firmly believe in their ability to cope with various challenges in prison work, and this positive self-perception motivates them to take proactive actions when facing stress and setbacks. According to Bandura’s [25] theory of self-efficacy, individuals decide whether to take action and what action to take based on their judgment of their own abilities. Employees with high GSES actively seek solutions when dealing with issues such as disciplinary violations by inmates or difficulties in educational reform. In the process of continuously trying and successfully solving problems, they not only strengthen their sense of self-efficacy but also enhance their resilience. Recent foreign studies further indicate that employees with high self-efficacy can more effectively mobilize their own resources and maintain a good psychological state when facing occupational stress, thereby enhancing resilience [29].

In a study conducted on prison staff in certain areas, scholars found that by offering targeted psychological training courses to enhance employees’ GSES, it is possible to effectively improve their psychological state and strengthen their resilience [31]. The study specifically pointed out that the psychological training content involved areas such as emotional management and problem-solving strategies. After the training, employees were able to apply their GSES more effectively when facing work pressures, thereby improving their resilience levels. This corroborates the findings of the present study, indicating that in a prison environment, cultivating employees’ positive coping abilities also plays a significant role in enhancing resilience.

Prison nurses’ proactive coping and self-efficacy directly affect work quality and stress adaptation: Nurses need to respond proactively in scenarios such as inmates’ refusal of medical treatment, sudden illnesses, and infection risks (e.g., personalized communication, emergency handling, standardized operations) [32, 33]. Core influencing factors include proficiency in professional skills, work experience, teamwork, and the effectiveness of vocational training. Nurses with high self-efficacy can make quick decisions in stressful situations—for example, efficiently performing emergency rescue operations and cooperating to maintain order when an inmate suffers from sudden heart failure—while those with low self-efficacy are prone to errors due to anxiety. Additionally, nurses’ proactive behaviors such as workflow optimization and risk prediction are all based on solid professional competence and occupational confidence.

### The relationship between resilience and job burnout and social support

However, it is noteworthy that this study found no significant direct correlation between resilience and job burnout or social support, which contrasts with findings from some prior studies [34, 35]. This result is closely tied to the sample’s uniqueness and the pandemic’s extreme working environment, requiring context-specific interpretation.

First, regarding resilience and job burnout: The sample’s high burnout prevalence (24.1%)—far exceeding general occupational groups [5] —stems from dual medical and prison management pressures, prolonged closed duty, and COVID-19 risks [1, 36]. Such intense, persistent stress surpasses resilience’s “buffering threshold“ [8], nullifying its protective effect, as seen in correctional staff research [19]. Additionally, the cross-sectional design limits insight into dynamic causal relationships; resilience may slow burnout progression [37] rather than affect current states, which is unreflected in high-baseline burnout data.

Second, for resilience and social support: Mismatched support supply and needs drive the lack of correlation. The PSSS scale measures family, friend, and other support [17], but closed management hindered external support delivery [1]. Internal prison support focused on logistics [37], while distinct ward needs (e.g., emotional/clinical support for Ward 2 [38], crisis intervention for Ward 3 [27] went unmet. Only job-specific social support predicts resilience [39], and untargeted support yields low perceived effectiveness.

Furthermore, measurement tools may contribute: MBI-GS emphasizes burnout’s emotional exhaustion, depersonalization, and reduced accomplishment [14], while CD-RISC focuses on resilience’s tenacity, optimism, and strength [18], with weak direct dimensional relevance—resilience may influence burnout via intermediate variables like coping styles [23]. PSSS measures “perceived support“ [17], and limited information exchange in closed environments may underestimate objective support, distorting results.

These negative findings reflect prison hospital staff’s unique circumstances under extreme conditions, not negating universal correlations but highlighting constraints from environmental intensity, support matching, and research design. Future longitudinal studies should explore dynamic resilience-burnout interactions, and targeted social support systems tailored to work area characteristics are needed to enhance effectiveness.

### Differentiated management and support strategies for prison zones

Targeting significant resilience differences among employees across prison zones, interventions can focus on work content optimization and team building. For high-pressure frontline zones like Zone 2 (managing incarcerated individuals with illnesses), a phased shift system may be adopted to balance work intensity and avoid prolonged stress. Meanwhile, professional psychological intervention teams should be introduced to provide regular counseling and stress relief training, helping employees address mental fatigue and emotional exhaustion. For Zone 3, its effective team-building and stress management experiences can be summarized into a replicable support model—such as strengthening staff mutual assistance and conducting humanized management training—for promotion to other zones. Administrative departments can leverage their resources to offer more efficient logistical and administrative support for frontline units, reducing their extra workload [39, 40].

### Paths to enhance positive coping and self-efficacy

Centering on the positive correlation between resilience, positive coping, and general self-efficacy (GSES), a targeted training system can be designed. On one hand, develop a “Positive Coping Skills Workshop” to train employees in proactive dilemma-solving strategies (e.g., problem-solving techniques, emotion regulation) through simulating typical stressful scenarios in prison work [41]. On the other hand, launch a “Self-Efficacy Enhancement Program” integrating Bandura’s self-efficacy theory, boosting staff confidence in tackling work challenges via successful experience sharing and role modeling. Additionally, foster an organizational culture encouraging positive coping across the prison by drawing on high-resilience zones’ practices, and incorporate positive coping behaviors into employee assessment and incentives to form a positive cycle [42].

### Precise construction of social support systems

Although this study found no significant correlation between resilience and social support, optimizing the precision and effectiveness of internal prison social support remains necessary. Conduct staff needs surveys to identify specific support requirements for different zones and positions in coping with job burnout—for example, Zone 2 employees may need more medical expertise and emergency response resources, while Zone 3 staff may prioritize psychological crisis intervention skills [43]. Based on these needs, establish a “hierarchical and classified support network” (including mutual aid groups, professional psychological counseling, and family support linkage mechanisms) to ensure resources accurately match needs and effectively enhance resilience [44, 45].

### Significance of this study

This study has both theoretical and practical significance. Theoretically, it supplements the research on resilience of Chinese prison staff—most existing studies focus on medical staff or teachers, while this study explores the unique influencing factors of resilience in the prison context (e.g., dual pressure of supervision and medical care in Ward 2, specialized training for mentally ill prisoners in Ward 3), enriching the empirical basis of resilience research in special occupational groups [46]. Practically, the study’s findings provide a basis for prison management to formulate targeted intervention measures: for example, the “nursing-staff co-management model” for Ward 2 and the “specialized training promotion plan” for Ward 3 can be directly applied to improve staff resilience and reduce burnout.

Compared with international studies, this study highlights the characteristics of Chinese prison management—e.g., the integration of prison supervision and medical care (Ward 2’s management model) is different from the separation of supervision and medical care in Western prisons [47, 48]. This provides a reference for cross-cultural research on prison staff resilience, as Sen et al. [49] pointed out that “cross-cultural differences in correctional systems affect the factors influencing staff resilience, and Chinese-specific research can promote the globalization of correctional psychology research”.

### Limitations of the study

Although the study has yielded valuable conclusions as mentioned above, there are still some limitations that need to be improved in future research.

### Sample limitations

This study focused only on certain wards and administrative departments of prisons in Chongqing Municipality, China. The sample scope is relatively narrow and may not fully represent the situation of all employees in the Chinese prison system. Prisons in different regions may exhibit different characteristics of job burnout and resilience due to regional culture and management models. Future research should expand the sample scope to include prisons from more regions to obtain more universally applicable conclusions.

### Single research method

This study mainly used questionnaire surveys and other quantitative research methods to collect data and analyze relationships. Although this approach can effectively obtain a large amount of data and conduct statistical analysis, it may not be sufficient for exploring some deep-seated causes and individual differences. Subsequent research could combine qualitative methods such as interviews and case studies to gain an in-depth understanding of the specific experiences and feelings of prison employees regarding job burnout and resilience from an individual perspective, in order to more comprehensively reveal the underlying mechanisms.

### Dynamic changes not considered

This study was based on data analysis from a specific period and did not fully consider the dynamic changes in job burnout and resilience of prison employees over time, changes in the work environment, policy adjustments, and other factors. Future research should conduct longitudinal studies to track the status changes of employees at different times, in order to more accurately grasp the development patterns of their job burnout and resilience. It is hoped that through the continuous improvement of subsequent research, a deeper and more comprehensive understanding of this field can be achieved, providing stronger support for prison management and employee development.

## Conclusion

This study explored the resilience of prison staff in Chongqing, China, revealing regional differences within prisons and associations with psychological factors. Resilience showed a significant positive correlation with positive coping strategies and general self-efficacy (GSES), but no significant direct association with job burnout was observed. This study has several limitations, including a geographically restricted sample, reliance on cross-sectional data, and lack of longitudinal tracking. Nevertheless, these findings may provide preliminary insights for prison administrators in comparable urban prison systems to design targeted resilience interventions, pending further replication studies with diversified samples and methods.

## Data Availability

Since the data and materials are related to the privacy of prison staff, they cannot be publicly available.

## Abbreviations

MBI-GS: Maslach Burnout Inventory-General Survey
EPQ: Eysenck Personality Questionaire
GSES: General Self-Efficacy Scale
PSSS: Perceived Social Support Scale
SCSQ: Simplified Coping Style Questionnaire
CD-RISC: Conner-Davidson Resilience Scale
